# Clinical Features of Dengue in a Large Vietnamese Cohort: Intrinsically Lower Platelet Counts and Greater Risk for Bleeding in Adults than Children

**DOI:** 10.1371/journal.pntd.0001679

**Published:** 2012-06-26

**Authors:** Trung Dinh The, Thao Le Thi Thu, Dung Nguyen Minh, Ngoc Tran Van, Hien Tran Tinh, Chau Nguyen Van Vinh, Marcel Wolbers, Tam Dong Thi Hoai, Jeremy Farrar, Cameron Simmons, Bridget Wills

**Affiliations:** 1 Hospital for Tropical Diseases, Ho Chi Minh City, Vietnam; 2 University of Medicine and Pharmacy of Ho Chi Minh City, Ho Chi Minh City, Vietnam; 3 Oxford University Clinical Research Unit, Hospital for Tropical Diseases, Ho Chi Minh City, Vietnam; 4 Centre for Clinical Vaccinology and Tropical Medicine, Oxford University, Oxford, United Kingdom; Pediatric Dengue Vaccine Initiative, United States of America

## Abstract

**Background:**

As dengue spreads to new geographical regions and the force of infection changes in existing endemic areas, a greater breadth of clinical presentations is being recognised. Clinical experience suggests that adults manifest a pattern of complications different from those observed in children, but few reports have described the age-related spectrum of disease in contemporaneous groups of patients recruited at the same geographical location.

**Methodology/Principal Findings:**

Using detailed prospectively collected information from ongoing studies that encompass the full spectrum of hospitalised dengue cases admitted to a single hospital in southern Vietnam, we compared clinical and laboratory features, management, and outcome for 647 adults and 881 children with confirmed dengue. Signs of vascular leakage and shock were more frequent and more severe in children than adults, while bleeding manifestations and organ involvement were more common in adults. Additionally, adults experienced significantly more severe thrombocytopenia. Secondary infection but not serotype was independently associated with greater thrombocytopenia, although with a smaller effect than age-group. The effect of age-group on platelet count was also apparent in the values obtained several weeks after recovery, indicating that healthy adults have intrinsically lower counts compared to children.

**Conclusions/Significance:**

There are clear distinctions between adults and children in the pattern of complications seen in association with dengue infection, and these depend partly on intrinsic age-dependent physiological differences. Knowledge of such differences is important to inform research on disease pathogenesis, as well as to encourage development of management guidelines that are appropriate to the age-groups at risk.

## Author Summary

Dengue is a common and potentially serious viral illness. Complications include plasma leakage from small blood vessels causing shock and dysfunction of the systems that control blood clotting, resulting in bleeding. The disease used to affect children predominantly, but in recent years, the number of adult patients has been increasing. As there is limited data describing the patterns of complications by age, we performed this study to compare clinical and laboratory features, management, and outcomes of the disease for over 1,500 children and adults with confirmed dengue recruited at the same time at a single hospital in the Southern Vietnam. We found that plasma leakage and shock were more common and severe in children than adults, while bleeding and organ dysfunction were more frequent in adults. Adults had lower platelet counts throughout the illness course as well as at a follow-up visit several weeks after recovery. Platelets are a crucial element in controlling bleeding, and the intrinsically lower counts in adults compared to children may contribute to the greater risk for bleeding in this patient group. Knowledge about differences in the patterns of dengue-related complications between children and adults should help clinicians to diagnose and treat patients more effectively.

## Introduction

Dengue is emerging as the most important mosquito-borne viral disease in the world. Infection with any of the four dengue viral (DENV) serotypes may result in asymptomatic infection or cause a variety of disease manifestations ranging from non-specific viral illness to a systemic vascular leak syndrome that is typically accompanied by thrombocytopenia, impaired haemostasis and liver dysfunction [1]. Shock due to severe plasma leakage occurs in a proportion of these patients, but other serious complications appear to be rare. The disease has spread rapidly across the tropical and sub-tropical world , with current estimates indicating around 50 million symptomatic infections occur annually [1].

In Southeast Asia, the epicenter of the current pandemic, dengue used to affect children predominantly but recently increasing numbers of young adults have required hospitalization [2], [3], [4]. One plausible explanation for this trend could be a reduction in the force of infection due to socioeconomic development and/or improved vector control such that fewer people are exposed during childhood leaving a larger reservoir of susceptible adults [5]. Alternatively lower birth and death rates in transitioning economies may decrease the flow of susceptible individuals into the population and increase the longevity of immune individuals, increasing the likelihood that an infectious mosquito will feed on an immune individual [6].

Reports describing the clinical picture in adults have only recently started to emerge, and suggest that unusual complications with high mortality such as acute liver failure, encephalopathy, myocarditis and acute renal failure are more common in this group than in children [2], [7]. In one study from Nicaragua signs of vascular leakage, shock and marked thrombocytopenia were seen less commonly with increasing age, although internal haemorrhage occurred more frequently in the adult subjects [8]. In a small prospective study from Sri Lanka, evidence of vascular leakage and shock were also seen more frequently in children compared to adults, although bleeding manifestations were similar [9], while in three small studies from Southeast Asia bleeding manifestations, thrombocytopenia and hepatic dysfunction were seen more commonly in young adults with the frequency of shock similar to the paediatric patient group [2], [3], [10].

Since 2004 we have been conducting large prospective descriptive studies of dengue encompassing the full spectrum of disease presenting to the Hospital for Tropical Diseases (HTD) in Ho Chi Minh City [11], [12], [13]. In this report we aimed to describe the patterns of disease, management strategies, and outcomes, comparing paediatric and adult cases recruited systematically into these studies during specified time-periods.

## Methods

### Patients and clinical methods

The following groups are eligible for recruitment after written informed consent is obtained from all patients or their parents/guardians: a) children 2–15 years old admitted to the Paediatric Intensive Care Unit (PICU) with clinically suspected dengue and overt complications; b) patients ≥15 years old with suspected dengue admitted to the Adult Intensive Care Unit (AICU) for any reason; c) children 5–15 years old or adults ≥15 years old admitted to the paediatric or adult infection wards with suspected dengue. Ethical approvals are obtained from the Scientific and Ethical Committee of the Hospital for Tropical Diseases and the Oxford University Tropical Research Ethics Committee UK. Eligibility criteria are deliberately broad to allow the study physicians to invite all potential subjects with suspected dengue to participate. However, the PICU is extremely busy and as study enrollment adds to staff workload children are eligible for recruitment only if complications severe enough to warrant intervention develop; patients who are admitted for close observation but receive no specific treatment other than maintenance fluid therapy are not recruited. Secondly, children less than 5 years old have a much lower risk for symptomatic dengue disease than older children, and as the study protocols request daily blood samples we elect not to subject young children admitted to the infection wards with non-severe febrile illness that is unlikely to be dengue, to this discomfort. Demographic information, clinical history and examination details are recorded on specific case report forms (CRFs) and all subjects are seen once daily until discharge by experienced study physicians trained to record all events occurring in the previous 24 hours directly into the CRF. Diagnostic samples for dengue serology/virology are obtained at study enrolment and hospital discharge. Management remains in the hands of the ward clinicians following hospital policies and WHO guidelines. All participants are invited for review several weeks after discharge when a final diagnostic blood sample is obtained.

For this comparison we included all patients recruited on the ICUs during the two years between September 2006 and September 2008 and all patients recruited in the infection wards during the year 2007. HTD is the main referral hospital for adults with dengue for southern Vietnam but receives few paediatric referrals. Since referred patients are likely to have more severe disease than direct admissions we considered these two groups separately and structured the comparisons between the age groups to take into account the route of admission. Definitions for all clinical complications assessed are presented in Table 1.

**Table 1:** Definitions for complications seen in confirmed dengue cases.

Complication	Definition
**Dengue shock syndrome**	Hypotension for age or narrowing of the pulse pressure ≤20 mm Hg, with impaired peripheral perfusion [1], if considered to be caused by plasma leakage not bleeding, and requiring volume resuscitation
**Bleeding severity**	Bleeding severity was coded retrospectively at discharge into four categories [12], [13]:-1. no clinical bleeding detected throughout observation2. minor skin bleeding only (petechiae or bruising at venepuncture sites)3. mild/moderate mucosal bleeding (no intervention required) with or without minor skin bleeding4. severe bleeding - bleeding requiring intervention (eg transfusion, nasal packing), or bleeding into a vital organ (e.g. intracranial bleeding)
**Fluid overload**	Respiratory distress due to significant ascites and/or pleural effusions, without evidence of any other respiratory pathology such as pneumonia
**Encephalopathy**	Any degree of mental alteration (Glasgow coma score ≤14)
**Acute liver failure**	Any degree of mental alteration with increased liver enzymes and a coagulopathy (demonstrated by an international normalized prothrombin ratio ≥1.5) in patients without evidence of pre-existing cirrhosis [25]
**Severe liver dysfunction**	Either acute liver failure (as above), or new onset of jaundice with significant increase in transaminase levels (≥300 U/L) considered to be due to dengue, without evidence of any other pathology such as acute viral hepatitis
**Acute renal failure**	Increase in serum creatinine at least 3 times baseline, or serum creatinine ≥4 mg/dl with an acute increase of >0.5 mg/dl [26]

### Laboratory investigations

Platelet counts and haematocrit measurements were performed at least once daily on all study patients in the hospital laboratory, with additional haematocrit measurements performed on the ICUs as frequently as required for clinical management. Overall percentage haemoconcentration was calculated as previously described [12], [13]. Liver function tests and coagulation screening tests were only carried out on adults during this time and the findings have been reported elsewhere [13]. Dengue diagnostic capture IgM and IgG ELISA assays were performed using paired enrolment and convalescent specimens and reagents provided by Venture Technologies (Sarawak, Malaysia) [14]. Using the enrolment specimen, DENV RT-PCR was carried out as previously described for a) all paediatric patients (who were generally admitted early) and b) all adult patients enrolled within the first 5 days of illness, and those with negative or inconclusive serology [14]. If both serology and RT-PCR were negative or inconclusive, assays for non-structural protein 1 (NS1) were performed using Biorad Platelia™ Dengue NS1 Antigen kits following the manufacturer's instructions. Patients were diagnosed to have confirmed dengue if the RT-PCR and/or NS1 results were positive, seroconversion was documented on paired serology, or dengue specific IgM was identified in a patient with a typical clinical syndrome. To define immune-status the ratio of IgM to IgG on or after day 6 of illness was used; a ratio ≥1.78 defined the infection as primary and one ≤1.2 as secondary [15]. Patients with ratios between these values, or in whom the results of enrolment and convalescent specimens differed, were considered unclassifiable.

### Statistical analysis

Data on clinical characteristics and laboratory results were compared between different patient groups using the Chi-square test or Fisher's exact test for categorical variables, and the Mann Whitney test or the Cuzick test for trend for continuous variables. When comparing platelet counts between children and adults we used multiple linear regression to assess the effect of covariates including age-group (age <15 years versus age ≥15 years, sex, infecting serotype (DENV 1–4 or unknown), and immune status (primary or secondary infection or unknown status). All statistical computations were carried out using SPSS Version 14 (Chicago, Illinois, USA) and Stata-SE Version 8 (Texas, USA).

## Results

During the selected time-periods a total of 947 children and 738 adults with suspected dengue were enroled into the various studies at HTD. Among them, 881/947 (93%) children and 647/738 (88%) adults were confirmed to have dengue on one or more of the diagnostic assays. The infecting serotype was identified in 450/881 (51%) children, and in 207/551 (38%) adults in whom DENV RT-PCR was performed. Serotype distribution was similar in the two populations: DENV-1 was detected in 254/450 (56%) children and 108/207 (52%) adults; DENV-2 in 102 children (23%) and 65 adults (31%); DENV-3 in 93 children (21%) and 30 adults (14%); and DENV-4 in 3 children (1%) and 4 adults (2%). Two children had mixed infections. The following report focuses only on patients with laboratory confirmed dengue. Co-morbidities were uncommon, and were distributed across the severity groups; 1 child and 8 adults had a history of asthma, 2 children had epilepsy and one child had haemophilia, 2 adults had thyroid disease, one had diabetes mellitus and three each had valvular heart disease and hypertension. All patients with co-morbidities survived. Notably, all 3 pregnant adult females in the study developed dengue shock syndrome (DSS).

### Clinical description of patients requiring intensive care

**Figure 1:**
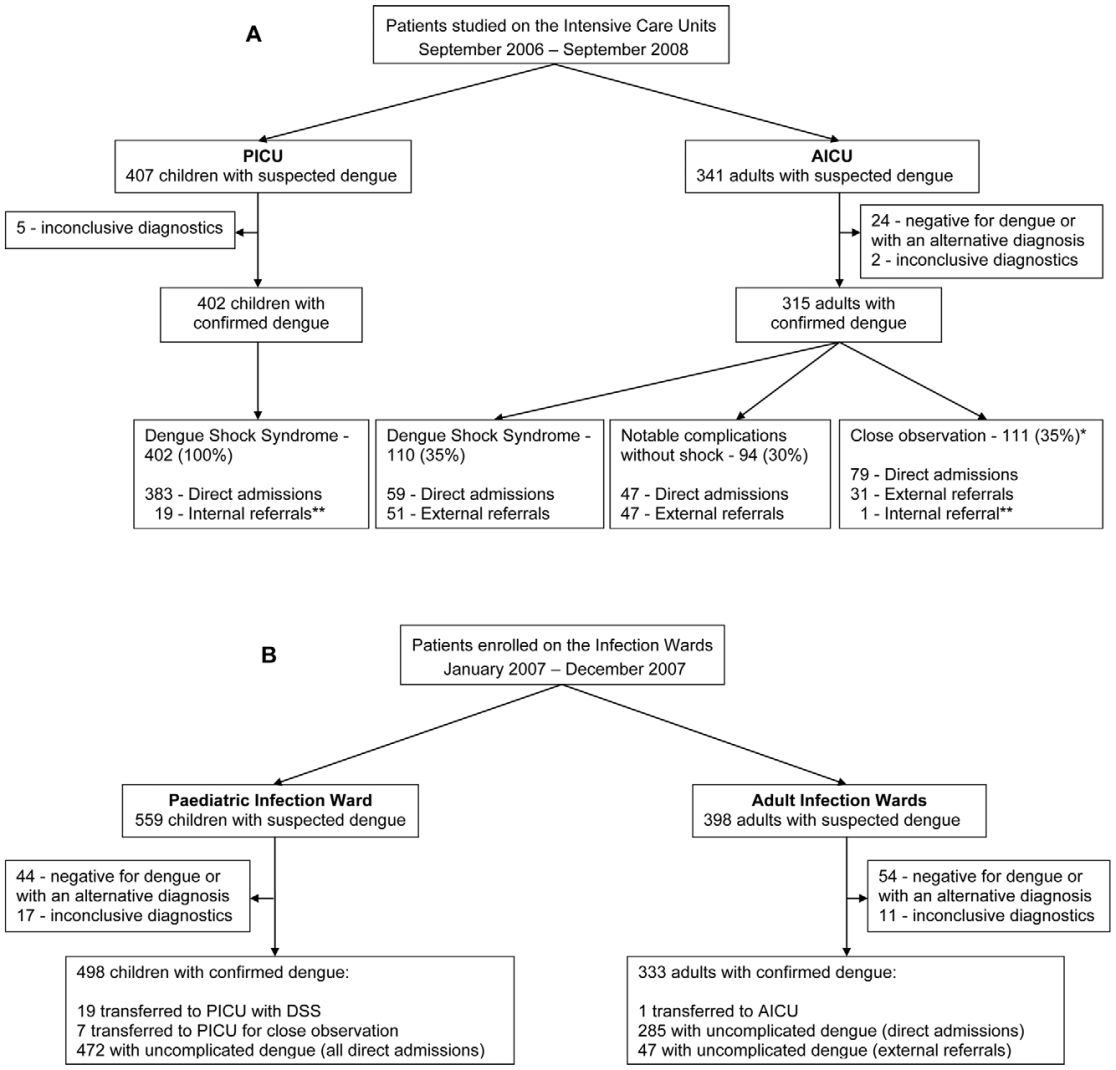
Profile of patients studied on the intensive care units (Panel A) and the infection wards (Panel B). * These 111 patients were admitted to AICU for close observation but did not go on to develop shock or other complications. Due to workload constraints children admitted to PICU with suspected dengue were only enrolled into the observational study if they developed overt complications. ** Note that these patients were recruited into the study in the infection wards and subsequently transferred to PICU/AICU.

Between September 2006 and September 2008, we recruited 388 children admitted directly to PICU with suspected dengue and hypovolaemic shock (>95% of all such cases) plus a further 19 children who were recruited into the study in the paediatric infection ward and subsequently transferred to PICU with clinical DSS (Figure 1). There were no external referrals from other facilities during the two-year period. Of the 407 children studied, 402 (99%) were confirmed to have dengue, and the remaining 5 had inconclusive results. Immune status could be reliably determined in 325/402 (81%) of cases, of whom 18 children with DSS (6%) had primary infections (DENV-1 = 13; DENV-2 = 1; unknown serotype = 4).

During the same two-year period 341 adults admitted to AICU with suspected dengue were also recruited (90% of all suspected dengue admissions to AICU), of whom 315 (92%) were subsequently confirmed to have dengue. Among the remaining 26 patients, 24 had negative dengue diagnostics and/or an alternative final clinical diagnosis (idiopathic thrombocytopenic purpura, septic shock, non-dengue encephalitis, hantavirus infection) and 2 patients had inconclusive dengue diagnostics. Only 186/315 (59%) of the adults with confirmed dengue were admitted directly to HTD with the remaining 129 (41%) being external referrals from other facilities. 110/315 (35%) of the adults studied in AICU had DSS (59 direct admissions and 51 external referrals), 94 (30%) experienced a variety of other complications (47 direct admissions and 47 external referrals), and 111 (35%) were admitted for close observation (79 direct admissions, 31 external referrals, 1 internal referral) but ultimately recovered without developing overt complications (Figure 1). Immune status could be determined in 260/315 (83%) of the patients in AICU, only 16 of whom had primary disease, with only 1 case identified among the patients with shock.

**Patients with DSS:** Table 2 presents a summary of key clinical features, management, and basic laboratory findings, comparing children and adults admitted directly to the two ICUs. Over the two-year period almost 7 times as many children as adults were admitted directly with DSS. The median age for paediatric patients was 9 years. Most adults were in their twenties or early thirties with no patient older than 37 years. Timing of onset of shock was similar in the two groups. Notably a number of patients (12% of children and 15% of adults) were still febrile at the time of onset of shock. Vomiting, abdominal pain and headache were more common among adults at presentation (p = 0.003, p<0.001 and p<0.001 respectively, χ^2^ test). All adults experienced some clinical bleeding, primarily mild/moderate mucosal bleeding, while one third of children had no bleeding and over 50% manifested only minor skin bleeding during the disease course. Eight children and 3 adults had severe bleeding, all in association with profound shock, and 5 children and 1 adult required blood transfusion.

**Table 2:** Key clinical features, therapeutic interventions, and laboratory findings, comparing children and adults with dengue shock syndrome managed in intensive care.

	Patients with DSS admitted directly		p*	Referred patients with DSS - Adults only (n = 51)
	Children (n = 402)	Adults (n = 59)		
**Characteristics reported or observed at enrolment** **				
Age, years	9 (3–14)	19 (15–32)	N/A	21 (15–34)
Male sex	220 (55)	29 (49)	0.42	20 (39)
Day of illness at shock	5 (4–6)	5 (4–6)	0.26	5 (3–6)^c^
Fever ≥38°C at shock	47 (12)	9 (15%)	0.43	-
Headache	109 (27)	52 (90)^a^	<0.001	42 (84)^a^
Vomiting	254 (63)	49 (83)	0.003	40 (80)^a^
Abdominal pain	205 (51)	46 (79)^a^	<0.001	40 (82)^b^
Fatigue	362 (90)	58 (98)	0.04	48 (94)
Rash	8 (2)	3 (5)	0.16	2 (4)^a^
BP unrecordable or PP≤10 mmHg at shock	60 (15)	11 (19)	0.46	-
**Summary of clinical features and complications observed during disease course**				
Hepatomegalyδ	365 (91)	43 (73)	<0.001	40 (78)
Clinical pleural effusion and/or ascites	147 (37)	22 (37)	0.91	26 (51)
Overall bleeding severity:-				
- No bleeding	131 (33)^a^	0	<0.001***	0
- Skin bleeding only	207 (52)^a^	21 (36)		9 (18)^a^
- Mild/Moderate mucosal bleeding	55 (14)^a^	35 (59)		30 (60)^a^
- Severe bleeding	8 (2)^a^	3 (5)		11 (22)^a^
Recurrent shock	147 (37)^c^	5 (8)	<0.001	27 (61)^d^
Severe liver dysfunction	0	0	-	2 (4)
Acute renal failure	0	0	-	2 (4)
Encephalopathy	1 (<1)	1 (2)	0.24	7 (14)
Length of hospital stay, days	3 (3–6)	5 (3–8)	<0.001	5 (1–16)
Death	3 (<1)	1 (2)	0.42	4 (8)
**Summary of treatment given**				
Total IV fluid given for shock (ml/kg)	114 (70–158)^c^	75 (45–124)	<0.001	91 (41–208)^e^
Colloid used	197 (49)^c^	8 (14)	<0.001	27 (61)^d^
Total colloid volume given (ml/kg)	17 (9–61)	12 (8–24)	0.05	17 (5–57)^b^
Whole blood/packed cells transfusion	5 (1)	1 (2)	0.56	8 (16)
Platelet transfusion	0	1 (2)	0.13	7 (14)
Inotropes used	19 (5)	1 (2)	0.49	9 (18)
Diuretics used	79 (20)	3 (5)	0.006	10 (20)
**Summary of key laboratory findings**				
Day of illness of maximum haematocrit****	5 (4–7)^b^	5 (4–6)	0.18	6 (4–7)^a^
Maximum haematocrit****	50 (44–56)^b^	51 (44.5–64.2)	N/A	47.4 (38.9–61.1)^a^
Percentage haematocrit change**** §	33 (17–58)^f^	26 (9–49)	<0.001	22 (2–57)^a^
Haematocrit change ≥20%****	351 (92)^f^	39 (70)	<0.001	20 (53)^a^
Platelet nadir, ×10^9^/L	32 (11–73)	18 (9–49)	<0.001	15 (6–43)^b^

Data are presented as number (percentage) for categorical variables and median (90% range) for continuous variables.

*: p value for comparisons between children and adults admitted directly with shock (Chi-square test or Fisher's exact test for categorical variables, and Mann Whitney test for continuous variables).

**: Age-dependent features such as pulse and respiratory rate are not presented.

***: Overall Chi-square test.

****: Haematocrit values are likely to be affected by severe bleeding during the acute illness, and thus percentage haemoconcentration was not calculated for patients with severe bleeding. For these variables the denominators are 393, 56 and 39 cases in the three patient groups respectively. Absolute values are age-dependent and were not compared statistically.

**δ:** Liver palpable below the costal margin in the mid-clavicular line.

**§:** Percentage change between the maximum recorded haematocrit between days 3 and 8 of illness compared to a baseline value obtained before day 3 of illness or at the follow-up visit. If neither was available the local population mean for age and sex was used as the baseline.

Missing data for.

a = 1 patient,

b = 2 patients,

c = 4 patients,

d = 7 patients,

e = 8 patients,

f = 10 patients.

Initial shock severity was similar in terms of the proportion with no recordable blood pressure or with very narrow pulse pressure (≤10 mmHg) at presentation. However adults experienced recurrent shock much less frequently than did children, indicated by the need for colloid resuscitation in only 8 (14%) adults as compared to 197 (49%) children (p<0.001, χ^2^ test). Adults were usually successfully managed with smaller volumes of fluid than children. Approximately one third of all patients developed clinically detectable pleural effusions or ascites, but only 3 (5%) of adults compared to 79 (20%) of children required diuretics. Three children (2 prolonged shock and severe bleeding, 1 prolonged shock with encephalopathy) and 1 adult (prolonged shock with encephalopathy) died despite full supportive care. All others made a full recovery.

Data for the 51 externally referred adults with DSS are also presented in Table 2 and clearly demonstrate that these patients were more severely ill than those admitted directly to AICU. Almost 60% of this group experienced recurrent shock and 11 patients (22%) developed severe bleeding. Clinical pleural effusions and ascites were common with 12/51 (24%) developing respiratory distress. Five patients required assisted ventilation (4 with fluid overload, and 1 with encephalopathy and pneumonia), while the remainder were managed successfully with oxygen and diuretics. Two patients had severe liver dysfunction demonstrated by jaundice and very high transaminase levels, and another two patients developed acute renal failure likely due to prolonged shock. A total of 7/51 (14%) of these externally referred adults became encephalopathic, 5 in association with severe bleeding. Four (8%) of the 51 patients died - 3 due to severe bleeding and fluid overload and 1 due to encephalopathy.

**Patients with other notable complications without shock:** Among the 94 patients admitted to AICU without DSS but with other notable complications, the clinical pattern was similar among direct admissions and external referrals, although the latter group tended to be more severely ill. There were 28/47 externally referred patients (60%) with severe bleeding (among them 3 cases with acute liver failure, 1 case with severe liver dysfunction, 2 cases with encephalopathy and intracranial bleeding demonstrated on CT scan, 1 case with a bleeding peptic ulcer, and the remaining 21 cases with no underlying cause detected), 14 patients (30%) with mucosal bleeding that settled without intervention, 2 patients with severe liver dysfunction but only skin or mild mucosal bleeding, and 1 case each with isolated encephalopathy, haemoglobinuria, and profound thrombocytopenia. Among the 47 direct admissions, there were 12 patients (26%) with severe bleeding (among them 2 cases with associated liver and renal failure, and 1 case with a bleeding peptic ulcer), 27 patients (57%) with mucosal bleeding that settled, 3 patients with syncope, 2 patients with haemoglobinuria and 1 case each with isolated encephalopathy, severe liver dysfunction, and profound thrombocytopenia. One of the externally referred patients with acute liver failure and severe bleeding died, but the remaining patients recovered.

**Patients admitted for observation:** A total of 111 patients were admitted to AICU for close observation, of whom 64 (58%) had transiently narrowed pulse pressure without evidence of impaired peripheral perfusion. None of these patients went on to develop clear signs of shock and all were discharged well without specific interventions other than maintenance fluid therapy. This group of adults appears to be of intermediate severity, with median platelet nadirs and percentage haemoconcentration significantly more abnormal than those of the adults managed on the infection wards but significantly less deranged compared to the adults admitted directly to AICU with DSS (data not shown). Although 270 children with suspected dengue were admitted to PICU for close observation during the same period, none developed shock or any other complications and thus were not recruited, as per the PICU research protocol.

### Clinical description of patients managed throughout on the infection wards

During 2007 we recruited 559 children with suspected dengue (approximately 25% of all such children as judged from the ward discharge records) and 398 adults with suspected dengue (approximately 10% of all such adults) admitted to the paediatric or adult infection wards at HTD. Of the 559 children, 498 (89%) were confirmed to have dengue, with immune status determined in 419 (84%), 130 primary and 289 secondary infections. Nineteen of these children (4%) developed DSS in hospital (see ICU section) and another 7 children were transferred to PICU for observation but did not develop shock. Of the 398 adults, 333 (84%) had dengue confirmed, among whom immune status was determined in 266 (80%), 30 primary and 236 secondary infections. Within the dengue-confirmed group 286 adults had been admitted directly to HTD while 47 were external referrals. Only 1 adult was transferred to AICU, a patient with known hyperthyroidism who developed rapid atrial fibrillation that responded to conventional treatment.

Table 3 presents the clinical features and key laboratory findings, comparing the 472 children and 285 adults admitted and managed throughout on the infection wards (hereafter referred to as uncomplicated dengue cases), with findings for the externally referred adults presented separately. Significantly more males than females were recruited in the paediatric ward. This was consistent with the observed male/female ratio observed among all admissions to that ward, but was not apparent in the male/female ratio for PICU admissions. Children were admitted to hospital approximately one day earlier than adults. However, adults were generally more symptomatic than children – most notably 70% complained of muscle pain compared to only 19% of children (p<0.001, χ^2^ test). Bleeding also occurred more frequently in adults. Almost half the children had no bleeding and minor mucosal bleeding was noted in only 10%, primarily epistaxis, compared to 46% of adults with mainly gum or minor upper gastrointestinal bleeding. In contrast more than 60% of children received some maintenance fluid therapy compared to 29% of the adults, either because of vomiting or high fever with inadequate oral intake.

**Table 3:** Key clinical features, therapeutic interventions, and laboratory findings, comparing children and adults with uncomplicated dengue managed on the infection wards.

	Patients with uncomplicated dengue admitted directly		p*	Referred patients with uncomplicated dengue Adults only (n = 47)
	Children (n = 472)	Adults (n = 285)		
**Characteristics reported or observed at enrolment** **				
Age, years	12 (7–14)	22 (15–34)	N/A	21 (16–39)
Male sex	303 (64)	132 (46)	<0.001	25 (53)
Day of illness	3 (2–5)	4 (2–6)	<0.001	4 (2–6)
Headache	325 (69)	260 (93)^d^	<0.001	44 (94)
Fatigue	424 (90)	255 (94)^f^	0.07	39 (83)
Muscle pain	90 (19)	190 (70)^f^	<0.001	33 (70)
Cough	76 (16)	66 (24)^c^	0.01	17 (36)
Vomiting	174 (37)	140 (50)^b^	<0.001	28 (60)
Diarrhoea	53 (11)	103 (37)^d^	<0.001	15 (32)
Abdominal pain	96 (20)	112 (41)^e^	<0.001	22 (47)
Rash	33 (7)^a^	37 (13)	0.006	4 (9)
Hepatomegalyδ	27 (6)	21 (7)^a^	0.36	7 (15)
**Summary of subsequent disease course**				
Day of illness at defervescenceξ	6 (4–8)^a^	6 (4–8)^c^	0.35	6 (4–9)
Overall bleeding severity:-				
- No bleeding	216 (46)^a^	46 (16)	<0.001***	3 (6)
- Skin bleeding only	209 (44)^a^	107 (38)		13 (28)
- Mild/Moderate mucosal bleeding	46 (10)^a^	132 (46)		31 (66)
Length of hospital stay, days	5 (3–7)	5 (2–7)	0.04	5 (3–8)
**Summary of treatment given**				
Any maintenance IV fluid given	292 (62)	84 (29)	<0.001	16 (34)****
**Summary of key laboratory findings**				
Day of illness of maximum haematocrit	6 (3–8)^a^	6 (4–8)^c^	0.003	6 (4–8)
Maximum haematocrit	43.9 (38.3–53.5)^a^	44.9 (38.8–53.9)^c^	N/A	45.1 (39.2–56.2)
Percentage haematocrit change§	14 (−2–37)^a^	10 (−3–32)^c^	<0.001	11 (−3–46)
Haematocrit change ≥ 20%	143 (30)^a^	49 (18)^c^	<0.001	9 (19)
Platelet nadir, ×10^9^/L	80 (30–178)^a^	42 (12–117)^c^	<0.001	40 (10–134)

Data are presented as number (percentage) for categorical variables and median (90% range) for continuous variables.

*: p value for comparison between children and adults with uncomplicated dengue admitted directly (Chi-square test or Fisher's exact test for categorical variables, and Mann Whitney test for continuous variables).

**: Age-dependent features such as pulse and respiratory rate are not presented.

***: Overall Chi-square test.

****: no information available prior to HTD admission.

**δ:** Liver palpable below the costal margin in the mid-clavicular line.

**ξ:** Day of defervescence was defined as the first day that the temperature dropped to 37.5°C and remained at or below this level subsequently.

**§:** Definition for percentage haematocrit change as in Table 2.

Missing data for.

a = 1 patient,

b = 3 patients,

c = 5 patients,

d = 6 patients,

e = 9 patients,

f = 13 patients.

### Key laboratory findings (Tables 2 and 3)

Normal haematocrit values are age-dependent, but the median maximum values recorded during the acute illness were similar, indicating greater increases in children than adults. Percentage haemoconcentration was also greater in children than adults in both the DSS and the uncomplicated dengue groups (both p<0.001, Mann Whitney test), and consequently the proportion of children with ≥20% haemoconcentration was also greater than among the adults (both p<0.001, χ^2^ test). The median day of illness on which the maximum HCT occurred was slightly later (day6) in the uncomplicated dengue patients than those with DSS (day 5).

**Figure 2:**
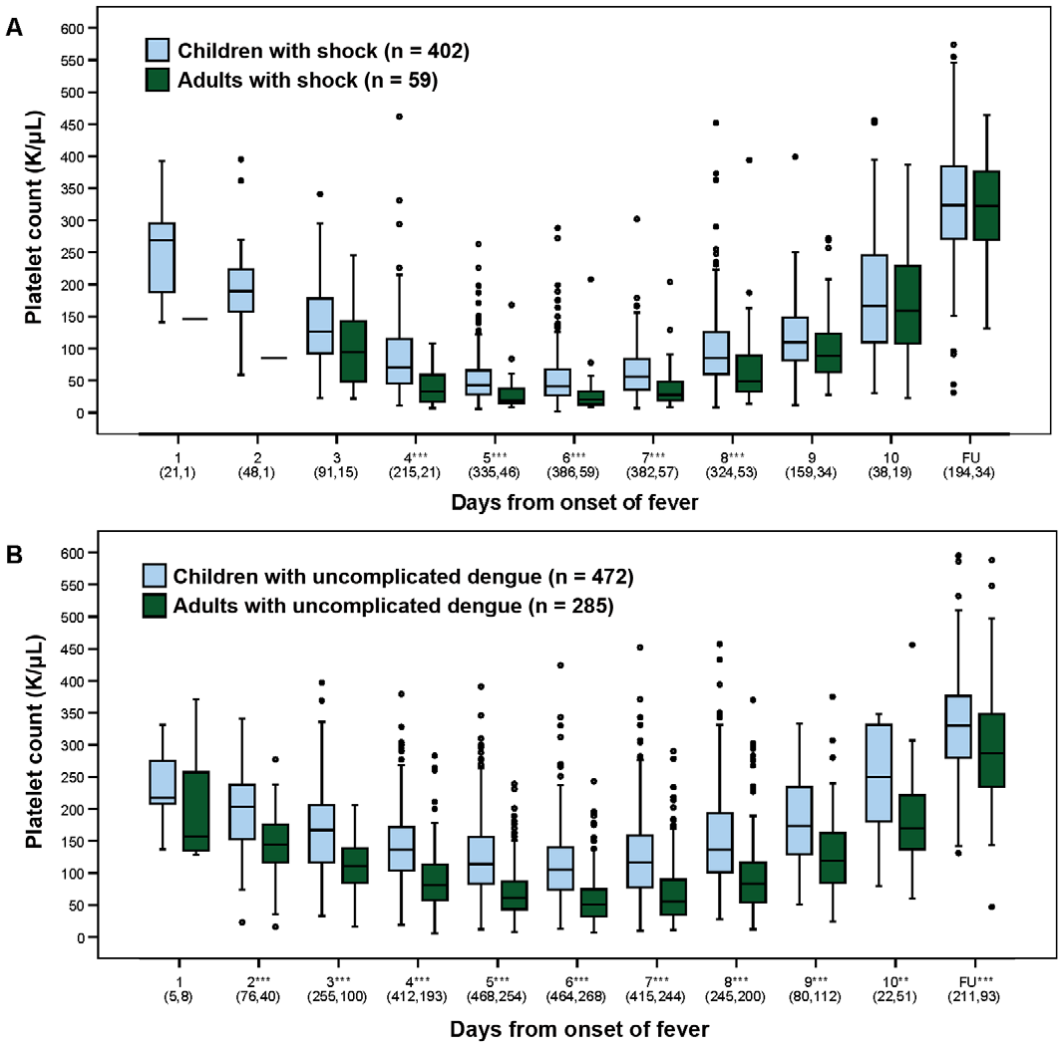
Daily platelet counts in children and adults with dengue. Box and whisker plots showing daily platelet counts during the course of illness and at follow-up for children and adults admitted directly to the relevant ICU with shock (Panel A), and for children and adults with uncomplicated dengue admitted directly to and managed throughout on the relevant infection wards (Panel B). Mean +/− SD day of illness at follow up for patients with DSS was 40+/−3 for children and 31+/−12 for adults, and for patients with uncomplicated dengue was 39+/−3 for children and 29+/−13 for adults. Boxes represent the median and interquartile values. Open circles indicate outlying/extreme values. The total number of patients included in each group each day is indicated on the X axis. The Mann-Whitney test was used to compare the daily platelet counts between children and adults, *** p<0.001 and ** p<0.01.

The platelet nadir occurred on day 6 of illness in both age-groups, with levels generally lower in those with shock than with uncomplicated dengue (Figure 2). Only 8 adults with shock - 4 with severe bleeding, and 4 with counts below 20×10^9^/L who required invasive procedures - and a further 13 adults with severe bleeding without shock, received platelet transfusions in addition to other blood products. No paediatric patient received a platelet transfusion. Considering patients directly admitted with shock, daily platelet counts were consistently lower among adults than children during the acute illness (p<0.001 for all daily comparisons between days 4–8, Mann Whitney test), although no difference was apparent at the follow-up visit. Among direct admissions with uncomplicated dengue, thrombocytopenia was consistently more severe in adults than children between days 2–10 of illness, and the adults' platelet counts were also significantly lower when reassessed at the follow-up visit after recovery (all p<0.01, Mann Whitney test). Multiple linear regression indicated that serotype had no significant effect on the platelet nadir (p = 0.53 for patients with uncomplicated dengue, p = 0.12 for patients with shock), but that there was an association with immune status i.e. secondary infection was an independent predictor of a lower platelet nadir (estimated effect size −22.0×10^9^/L (95% CI: −30.6 to −13.4), p<0.001 in the uncomplicated dengue group, and −13.2×10^9^/L (95% CI: −23.8 to −2.7), p = 0.01 in patients with shock). In addition, age-group had a pronounced effect on the platelet nadir (estimated effect of age ≥15 years was −35.5×10^9^/L (95% CI: −43.0 to −27.9), p<0.001 in patients with uncomplicated dengue, and −6.7×10^9^/L (95% CI: −13.2 to −0.2), p = 0.04 in patients with shock). Notably, age also significantly affected the platelet count at follow-up in the uncomplicated dengue group, with a larger effect size compared to that observed during the acute illness; the estimated effect of age ≥15 years (assessed by multiple linear regression adjusted for serotype and immune status) on follow-up platelet values was −41.7×10^9^/L (95% CI: −64.1 to −19.4), p<0.001. This relationship was not apparent for follow-up platelet values in the DSS group (estimated effect of age ≥15 years −6.5×10^9^/L (95% CI: −40.1 to 27.2), p = 0.7).

**Figure 3:**
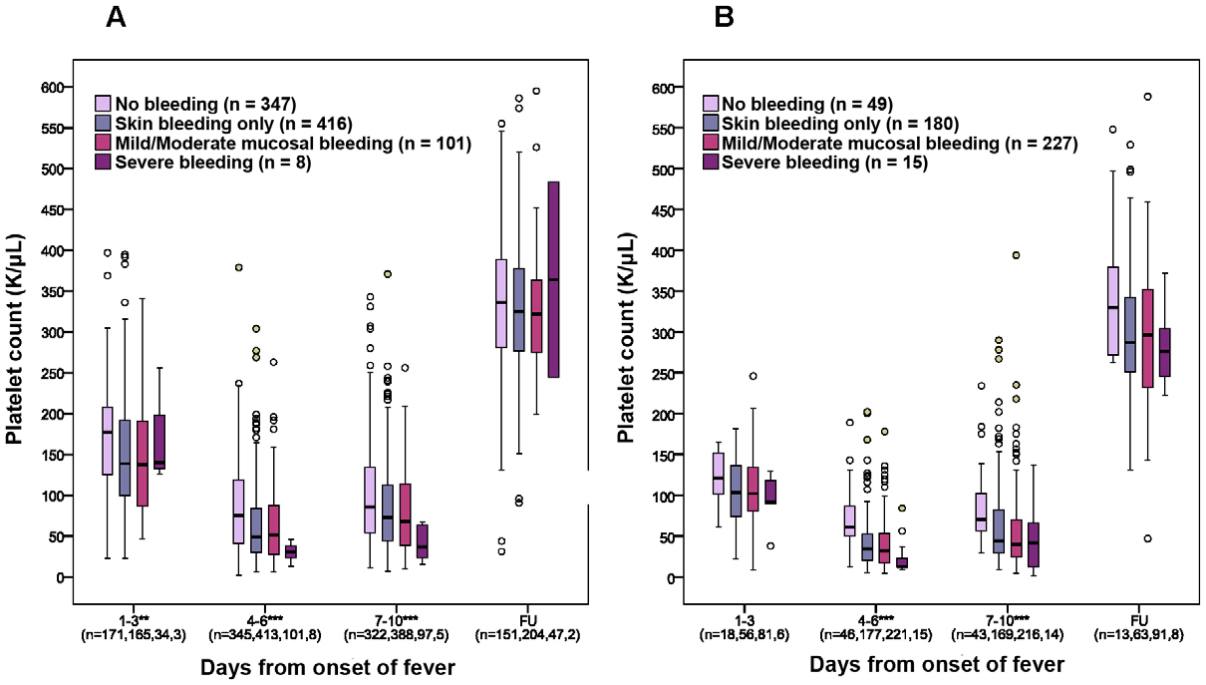
Associations between platelet counts and bleeding severity in children and adults with dengue. Box and whisker plots showing associations between the lowest platelet counts observed in specific time-periods (early febrile – days 1–3, critical – days 4–6 and convalescent periods – day 7–10) during the illness course and at the follow up visit, and the overall bleeding severity for all children (Panel A) and adults (Panel B) admitted directly to the hospital. Boxes represent the median and interquartile values. Open circles indicate outlying/extreme values. The total number of patients included in each group each time-period is indicated on the X axis. The Cuzick test for trend was used to compare the lowest platelet counts across the overall bleeding score in each time-period, *** p<0.001 and ** p<0.01.

**Figure 4:**
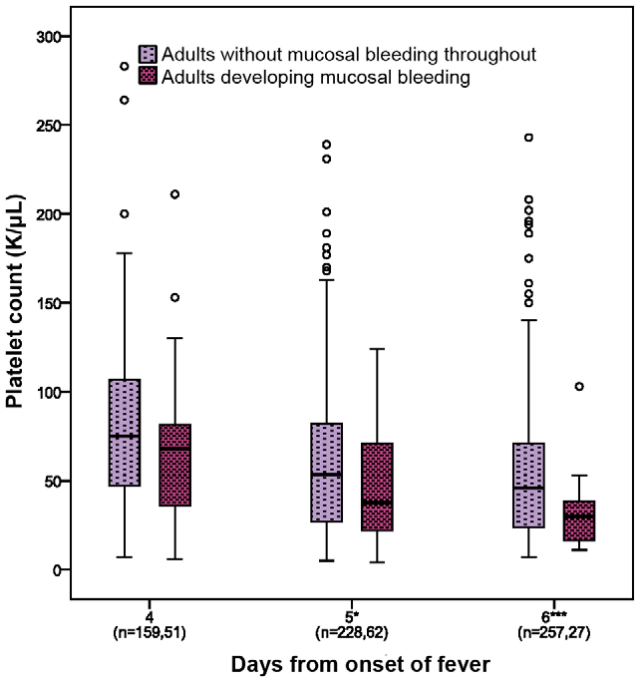
Platelet counts by day of illness for adults who did or did not develop mucosal bleeding. Box and whisker plots showing platelet counts on days 4, 5 and 6 of illness comparing adults who developed mucosal bleeding during the subsequent 24 hour period to adults who never developed mucosal bleeding throughout the illness course. Boxes represent the median and interquartile values. Open circles indicate outlying/extreme values. The total number of patients included in each group each day is indicated on the X axis. Patients in whom mucosal bleeding developed prior to admission to HTD are not included. The Mann-Whitney test was used to compare the platelet counts between the two groups on each day. *** p<0.001 and * p<0.05.

We also found significant correlations between lower platelet counts and the overall severity of bleeding graded at discharge. Figure 3 presents the lowest platelet counts recorded during specified time-periods during the illness course according to bleeding severity, for the children and adults separately. The majority of patients who developed mucosal bleeding were adults, with the onset usually between days 4–6 of illness. To assess relationships between platelet counts and onset of mucosal bleeding in more detail in this group, we compared the most recent counts measured prior to development of any mucosal bleeding (≤24 hours) with counts measured at the same time in the disease evolution for patients who never developed mucosal bleeding (Figure 4); the counts were significantly lower in those who developed mucosal bleeding on days 5 and 6 of illness (p = 0.03 and p<0.001, respectively) and borderline significant for day 4 (p = 0.06, all Mann-Whitney test).

## Discussion

The availability of detailed prospectively-collected observational data on a large number of dengue patients admitted to a single institution in an endemic region permitted this examination of the clinical patterns of dengue disease by age. We found that although the overall clinical picture was similar in children and adults some clear differences were apparent, particularly in the pattern of complications seen. Admission with shock and development of recurrent shock were more common in children than adults, and plasma leakage was more profound. In contrast bleeding manifestations and organ involvement were more common and more severe in adults. Thrombocytopenia was also more severe in adults, but this likely reflects similar disease-associated effects superimposed on intrinsically lower normal platelet values in adults compared to children.

Health-seeking behavior and local admission policies and referral systems may influence observed disease patterns, particularly in a hospitalized population. For example the male excess among children with uncomplicated dengue has been documented previously and may reflect parental health-seeking behavior [4], [16]. HTD admits around 8–10,000 patients with suspected dengue annually. Although it functions as the main provider of inpatient care for local adults with dengue as well as being a referral centre for severe adult disease, local children may be taken preferentially to one of two paediatric hospitals in the city and HTD receives few regional paediatric referrals. Therefore we focused the main analysis on direct admissions from the local area in order to better reflect patterns of symptomatic disease by age.

Our findings support the generally accepted view that severe vascular leakage is more common in children [8], [17]. Age is known to influence intrinsic vascular permeability, with children demonstrating a lower threshold for leakage than adults [18]. Similarly, homeostatic mechanisms aimed at minimizing cardiovascular decompensation in the face of increased permeability are less well developed in children. However it is important to note that the population affected by dengue in Vietnam, a hyper-endemic country, is relatively young - the oldest patient in the study was 57 years old and the oldest DSS case was 37 years old. Secondary infection is well recognised as a risk factor for severe disease; 6% of the paediatric shock cases had primary infections compared to 30% of the uncomplicated paediatric cases, while only 6% of adults overall had primary disease with a single patient (aged 18 years) identified among the shock cases. Reports from Taiwan and Singapore, where endemicity is lower than Vietnam and secondary infections likely to occur later in life, indicate that infections occurring in elderly patients with co-morbidities are at greater risk for severe complications and death [19], [20].

Skin and mild to moderate mucosal bleeding were significantly more common in adults, and severe bleeding in the absence of DSS occurred only in adults. Although we cannot exclude the possibility that some local children with severe bleeding without shock were admitted to one of the two paediatric hospitals, informal communication with both centres indicates this presentation to be rare. In the majority of the adults manifesting severe bleeding without shock few specific antecedents (such as peptic ulcer disease) could be identified. Coagulation abnormalities for the adult patients have been reported previously [13], showing a general pattern of prolonged partial thromboplastin times with reduced plasma fibrinogen levels, but little change in the prothombin time or evidence of procoagulant activation. This pattern of abnormalities has also been described among children with dengue, with increasingly severe abnormalities seen in association with more severe vascular leakage [12], [21], and the evidence to date suggests that a typical “dengue coagulopathy” occurs alongside the well-recognized thrombocytopenia. Since coagulation tests were not performed systematically in the children involved in this analysis no direct comparisons by age were possible, but we were able to demonstrate that thrombocytopenia was more marked in adults than children; we documented significantly lower platelet counts in the adults throughout the evolution of the disease, with clear associations with the overall severity of both leakage and bleeding.

The development of bleeding events across the spectrum from mild skin bleeding to severe mucosal bleeding probably reflects a combination of dengue-induced abnormalities on a background of characteristics intrinsic to the individual; thus it is unlikely that the lower platelet counts in adults are directly responsible for the greater risk for bleeding per se in this group. The multiple linear regression analysis indicated that the platelet/age-group effect cannot be explained solely by the fact that adults had more secondary infections than children – adult age-group was an independent predictor of lower platelet counts for both severity groups during the acute illness and for the uncomplicated dengue patients at follow-up. The absence of an apparent effect at follow-up in the DSS patients may be explained by differences in the timing of these visits. Most adults attended for review about 4 weeks from illness onset, at a time when rebound thrombocytosis was likely still to be a factor, while children generally returned for review 1–2 weeks later (Figure 2). Rebound thrombocytosis is normally proportionate to the severity of preceding thrombocytopenia and may have confounded the age effect in the DSS patients. In support of this, follow-up platelet counts were significantly higher in adults with DSS compared to adults with uncomplicated dengue, (measured on average 29 days from illness onset), while no difference was seen in the follow-up platelet counts in the respective paediatric patient groups, (measured on average 40 days from illness onset, data not shown). Few data are available on platelet changes with age, but one European study that examined platelet counts in 500 healthy subjects also demonstrated a progressive decline in platelet numbers during childhood, leveling off at 18 years [22].

We also found that children were generally less symptomatic than adults in the febrile phase of uncomplicated dengue, although they were more than twice as likely to receive maintenance intravenous fluid therapy. Comparisons of management strategies between the groups are complicated by the fact that both general and dengue-specific fluid management guidelines are tailored to expected age-related requirements. However the favourable outcome in most cases, together with the fact that significantly greater haemoconcentration was demonstrated in the children despite their receiving considerably more fluid therapy overall than the adults, indicates that the interventions were likely proportionate to need. Over the years considerable experience has accumulated regarding fluid management of children with dengue, but as epidemiological patterns begin to change and more adults present with severe and complicated disease, it is crucial that suitable fluid management algorithms are developed that are relevant to the age-groups at risk. Additionally, given that profound but frequently asymptomatic thrombocytopenia is almost universal in adults with dengue, the lack of practical guidelines on the use of blood products needs to be addressed. Prophylactic platelet transfusions are becoming almost routine in some dengue-endemic countries, using arbitrary thresholds without evidence of benefit [23], [24]. Among this cohort of 1528 confirmed-dengue cases (of whom only 9 patients died) prophylactic platelet transfusions were given to just 4 patients, all of whom had profound thrombocytopenia and required invasive procedures, in addition to a total of 17 patients who had severe bleeding. In view of the associated clinical risks and the financial cost of prophylactic platelet transfusions it is essential that formal research studies are carried out before this becomes established as the standard of care.

In summary, in this large study comparing the clinical features of dengue between adults and children we have demonstrated that the complications experienced by the two groups tend to reflect age-related physiological norms. Children, who have intrinsically higher microvascular permeability tend to develop vascular leakage and DSS, while adults tend to experience bleeding complications or severe organ impairment. The higher frequency and greater severity of bleeding manifestations in adults is likely to be driven by a variety of factors including a) intrinsically lower baseline platelet counts b) a greater likelihood of experiencing a secondary rather than a primary infection and c) a greater propensity to have underlying chronic diseases that might predispose the subject to bleeding. Knowledge of the patterns of disease in different age-groups is helpful firstly to facilitate development of suitable management guidelines. Secondly, as dengue spreads to new geographical locations and the epidemiological picture diversifies, it is crucial that changing patterns of disease are recognized and global trends identified.

## Supporting Information

Checklist S1 **STROBE checklist.**
(DOC)
